# Supplementary figures and images for: DeepHE: Accurately predicting human essential genes based on deep learning
Source: PLoS Comput Biol. 2020 Sep 16;16(9):e1008229. doi: 10.1371/journal.pcbi.1008229 (PMC7521708; doi:10.1371/journal.pcbi.1008229)

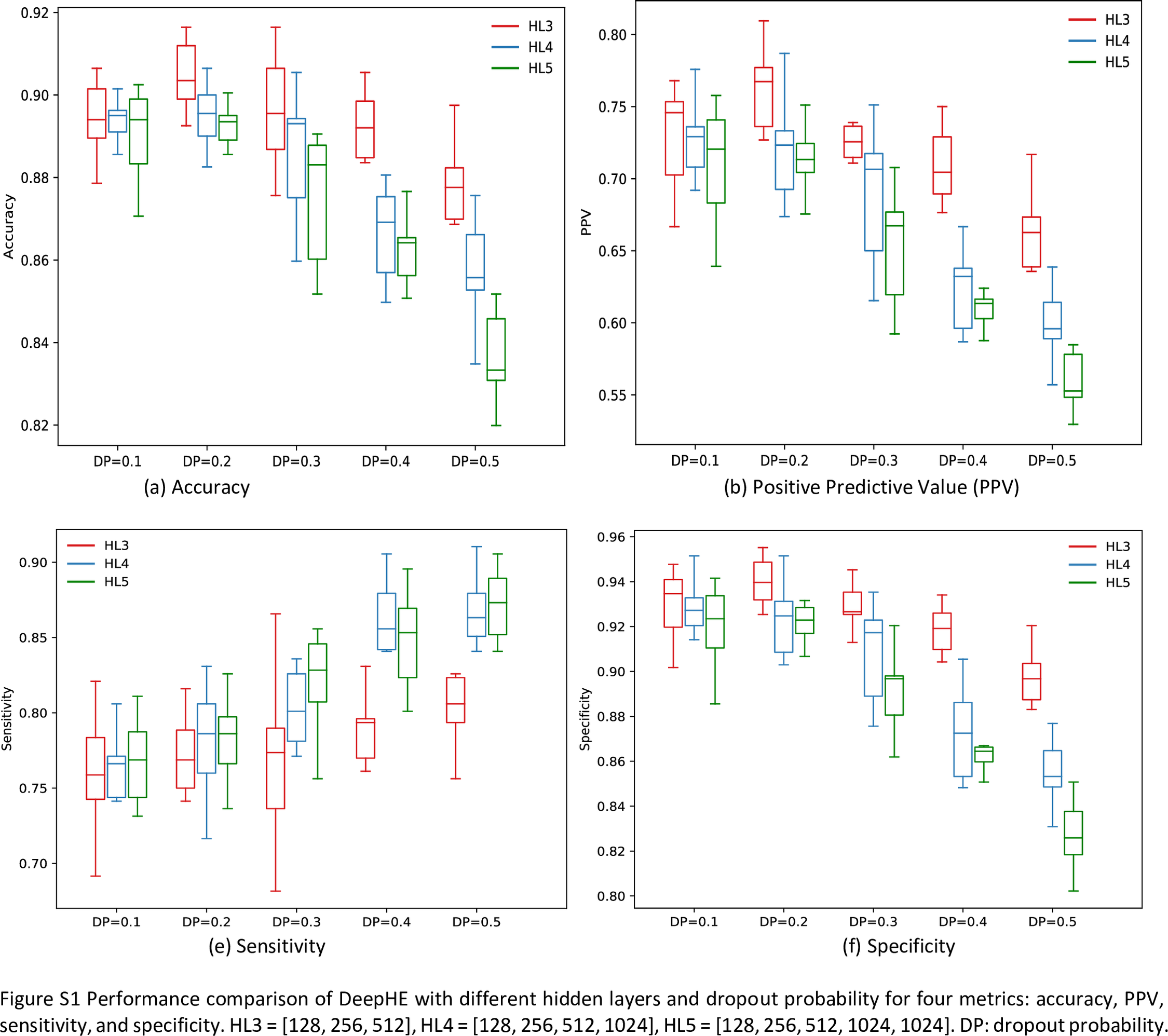

Supplement: S1 Fig — (TIF) [file pcbi.1008229.s001.tif]

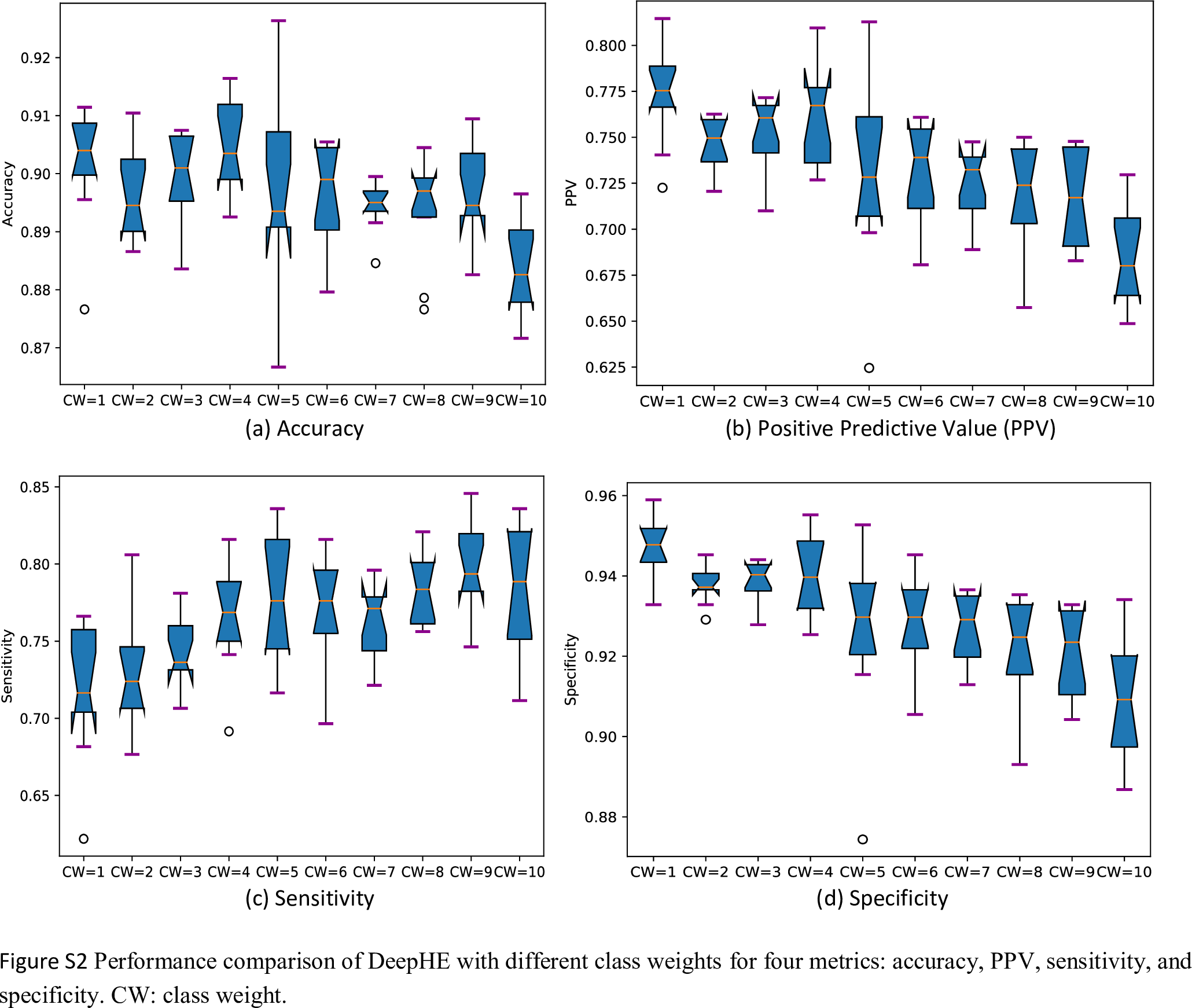

Supplement: S2 Fig — (TIF) [file pcbi.1008229.s002.tif]

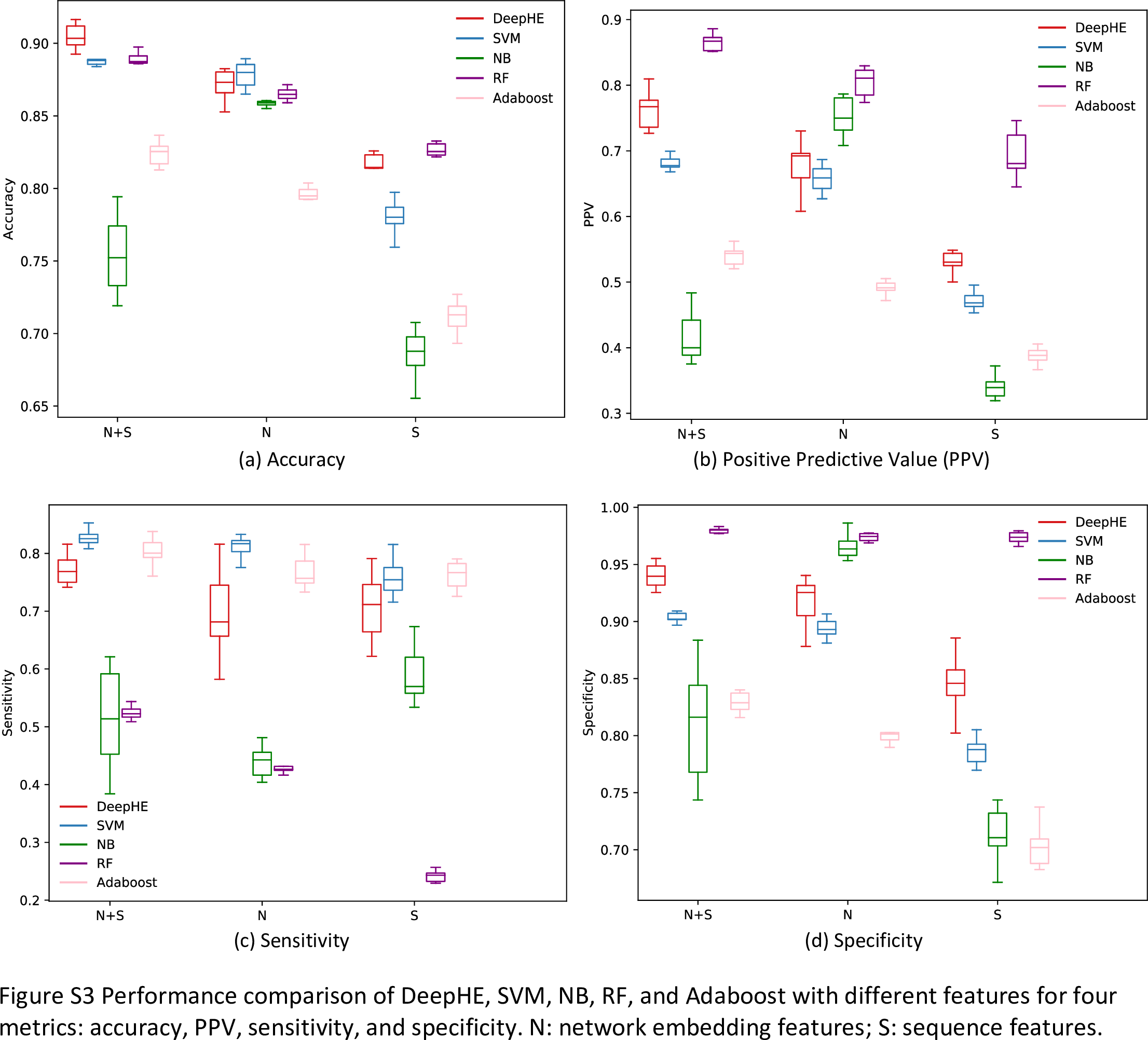

Supplement: S3 Fig — (TIF) [file pcbi.1008229.s003.tif]
